# Neuropsychological parameters in male offenders with substance use disorders

**DOI:** 10.3389/fpsyt.2024.1476920

**Published:** 2024-10-17

**Authors:** Fabian Hoffmann, Birgit Völlm

**Affiliations:** Clinic for Forensic Psychiatry, University Medicine Rostock, Rostock, Germany

**Keywords:** substance use, forensic psychiatry, neuropsychology, impulsivity, attention, psychomotor speed, executive function, decision making

## Abstract

**Introduction:**

The impact of cognitive functions on treatment outcomes in forensic psychiatric patients with substance use disorders is not well understood. This study investigates whether neuropsychological deficits, such as in attention, executive functions, and social-emotional cognition, are associated with impulsivity and criminal history.

**Methods:**

109 male patients with substance use disorders at the Clinic for Forensic Psychiatry in Rostock were screened using inclusion and exclusion criteria, with 30 consenting to participate. The tests included the Cambridge Neuropsychological Test Automated Battery (CANTAB) to assess cognitive functions in the areas of attention, psychomotor speed, social and emotional perception, and executive functions, with a particular focus on decision making, planning and problem solving. The Barratt Impulsiveness Scale (BIS-11) was used to measure impulsiveness.

**Results:**

Participants displayed significantly higher impulsivity levels on the BIS-11 compared to the general population and showed marked deficits in attention, psychomotor speed, and executive functions. There was a minimal correlation between impulsivity and cognitive performance, suggesting that impulsivity does not directly predict cognitive impairments. Notably, extensive criminal histories correlated with poorer cognitive performance, particularly in tasks requiring planning and problem-solving.

**Discussion:**

We found mixed support for the hypothesized associations between neuropsychological functions and criminal histories among patients with substance use disorders. While tasks related to planning and sustained attention showed clearer links, broader cognitive functions displayed inconsistent correlations. These findings emphasize the complexity of the relationship between cognitive deficits, impulsivity, and criminal history, highlighting the necessity for tailored assessments and rehabilitation strategies to enhance outcomes. Future research should focus on larger, longitudinal studies to validate these findings and refine therapeutic approaches.

## 1 Introduction

Forensic psychiatry is a specialty within psychiatry based on detailed knowledge of legal issues, criminal and civil justice, the purpose of which is the care and treatment of mentally disordered offenders, including risk assessment, risk management and the prevention of future delinquency (1).

In Germany, a distinction exists between prison sentences and forensic-psychiatric treatment orders in the German Criminal Code (StGB). While prison sentences apply to criminally responsible or partially responsible offenders, forensic-psychiatric treatment accommodates individuals with diminished or no responsibility, often due to mental health or substance use disorders (2–4). The aim of forensic psychiatry is to reduce the risk of reoffending by improving mental health (5). Unlike general psychiatry, courts decide on both admission and length of treatment, meaning patients cannot freely choose when or where they receive treatment (3).

In cases of substance use disorders, treatment is provided under Section 64 StGB, which pertains to offenders whose criminal behavior is primarily attributed to substance abuse. Placement in a detoxification facility is mandated if there is a risk of further significant unlawful acts due to the substance use disorder, and if treatment is expected to be successful (6).

A 2022 survey of German forensic psychiatric clinics found that 89% of patients treated under Section 64 StGB were male (7), with up to 50% not successfully completing treatment (8). Our own data from Rostock showed that among patients with substance use disorders discharged in 2009 and 2010, 34.1% of those attending forensic-psychiatric aftercare and 51.2% without aftercare had reconvictions within 2 to 4 years (9).

The literature reveals several risk factors of future criminal behavior. They are often categorized as static or dynamic, where the former are stable over time, and the latter are subject to change. Some of the static risk factors comprise of male gender, younger age, previous offending, and family criminality or violence (10–13). Dynamic risk factors include e.g. interpersonal conflicts, antisocial personality and companions, impulsivity, and importantly, substance abuse (10, 13–15) which stands out as an often replicated strong risk factor (16–18). One reason is that substance abuse can lead to disinhibition, making aggression more likely (14, 19). It can also negatively affect the ability to solve problems, potentially leading to conflict situations (19, 20).

Sariaslan et al. (17) analyzed data from over 47,000 released prisoners in Sweden and found that individuals with psychotic disorders and comorbid substance abuse had significantly higher risks of violent reoffending. Although neighborhood factors initially seemed to play a role, their influence diminished after controlling for confounding variables, emphasizing the stronger impact of individual factors such as substance abuse. Whiting et al. (18) reviewed evidence showing that individuals with psychiatric disorders, especially when combined with substance use, are at higher risk of violence. Substance abuse was identified as a strong predictor of violent outcomes, further reinforcing the need to prioritize its treatment in forensic settings. Garritsen et al. (19) examined forensic psychiatric patients and highlighted impulsivity, addiction, and exposure to risky social networks as key dynamic factors contributing to violent recidivism. They emphasized that addressing these factors, particularly substance abuse, is crucial to reducing reoffending rates.

The question arises which characteristics allow a reasonably reliable assessment of the prospects of treatment in offenders with substance use disorders (21). In a systematic literature review (22), in which predictors of the form of discharge (premature or regular) from 16 empirical studies from the period 1999-2019 were analyzed, findings were heterogeneous. The authors found that (with the exception of the factors personality disorder and psychopathy) diagnostic and psychometrically determined personal characteristics were less related to the discharge mode than static historical variables, in particular criminal history. Patients with a particular combination of factors such as early onset of delinquency, problematic social and/or occupational or educational background, accompanied by certain personality characteristics, showed a very high risk of premature ending (22). In a large empirical study including 777 patients released from German forensic addiction clinics type of discharge (regular discharge or premature treatment termination) was investigated. It was shown that structural setting variables such as the treating clinic or the court that ordered the initial detention were significantly associated with the type of discharge. A prognostic model incorporating these variables produced better results than a model based solely on person-related factors. Nevertheless, it must be noted that so far identified parameters explain only about one third of the variance of the therapy success (23). This shows that the current state of research cannot provide a sufficient answer to the question of the determinants of success or failure of treatment in forensic hospitals (22). In addition, predictors are conceivable that are not rooted in the socialization, personality or life history of the patients. These could have a significant influence on the course of forensic treatment completion (23) and future outcome. Here, cognitive functions come into focus of attention.

In non-forensic populations, various neuropsychological studies have provided insight into the neuropsychological functions of people with substance use disorders. To determine the long-term consequences of use of alcohol and illegal drugs, Yücel et al. (24) reviewed relevant studies from the last 20 years. It was suggested that chronic abuse of a wide range of addictive substances can impair neuropsychological functioning. There is consistent evidence that almost all substances of abuse have effects in the areas of attention, learning and memory, visuospatial abilities and executive functions. Most importantly they can lead to impairment of inhibitory control (also known as response inhibition), working memory and decision-making. Nevertheless, the author also noted that the pathways to addiction are complex and there are interindividual differences in patterns of substance use (e.g. duration, frequency, dosage, type). The fact that most studies in the review were cross-sectional means that it is not possible to determine whether the deficits found are a consequence of the specific drugs, are due to pre-existing vulnerabilities or a combination of both.

One of the key elements of drug addiction seems to be the lack of inhibitory control, including control of emotional, cognitive and behavioral responses (25–27). In a systematic meta-review conducted by Lee et al. (28) examining the neurocognitive functions central to impulsive-compulsive behaviors transdiagnostically across addictive behaviors, dependence on psychostimulants (i.e methamphetamine, cocaine, MDMA) or alcohol seemed to be more frequently associated with deficits in inhibitory control than cannabis dependence, whereas impulsivity was consistently absent. Tobacco use, by comparison, appeared to be associated with mild impulsivity. It has also been suggested that individual differences in the neurocognitive aspects of impulsivity (i.e. cognitive and motor disinhibition and impulsive decision-making) in individuals with substance use disorders are associated with differences in addiction treatment outcomes (29, 30).

Impulsivity is an important factor in adverse outcomes such as substance use or problem gambling. Research has demonstrated these negative outcomes are associated with both self-report and behavioral measures of impulsivity (31). Laboratory behavioral tasks assess what participants actually do in a given situation. This stands in contrast to participants’ reports of what they do over time and across situations, as assessed by questionnaires. Tasks capture more state-like phenomena than the traits assessed by self-report measures (32, 33). In a meta-analysis conducted by Sharma et al. (33), laboratory tasks that purport to measure a construct similar to trait impulsivity were reviewed. A meta-analytic principal-components factor analysis demonstrated that these tasks constitute 4 factors (Inattention, Inhibition, Impulsive Decision-Making, and Shifting). The literature has shown weak associations between these domains – behavioral tasks and self-report (33).

Focusing on offending populations, Dolan and Fullam, (34) examined 40 male personality disordered offenders with no significant co-morbid substance misuse or axis I pathology detained in a maximum-security hospital with regards to the relationship between psychometric (including the Barratt impulsivity scale (BIS)) and behavioral measures of impulsivity. As already established in other populations, behavioral measures did not correlate well with psychometric measures of impulsivity. Therefore it can be concluded that these measures assess different constructs. However, there is little evidence so far with regards to the relationship between these constructs in offenders with substance misuse.

In substance using offenders, several neuropsychological deficits have been shown. Some authors have suggested that substance-induced neurocognitive impairments increase the risk of engaging in criminal behavior (35). Craun, (36) has shown that increased alcohol use was associated with deficits in neurocognitive functioning, which were in turn associated with an increase in total number of convictions. Research has also revealed significant associations between neurocognitive deficits and aspects of criminal behavior in prisoners (37–39).

Focusing on violent offender populations, Romero-Martinez and Moya-Albiol, (40) conducted a literature review to examine the relationship between neuropsychological deficits due to abusive cocaine use and/or prenatal exposure and the expression of violence. Most studies focused on deficits in empathy and executive functions as important functions for social adjustment. The review concluded that deficits in decoding emotions (which could be explained by low sustained attentional capacity) may lead to a low ability to understand the feelings and thoughts of others, and impaired decision making, as these individuals do not properly assess the consequences of their actions. Risk of violence was also higher when the ability to verbalize emotions and think abstractly were severely impaired.

Comparing violent offenders, non-violent offenders and non-offending persons, Curtis et al. (41) examined whether the cognitive functioning of individuals with alcohol and other substance use histories presenting to a specialist addiction neuropsychology service differed according to their offending history. Data were extracted from 190 clients. Violent offenders demonstrated the lowest premorbid IQ out of the three groups, and a significantly higher proportion of violent offenders presented with impaired divided attention and impaired cognitive inhibition compared to non-violent offenders.

In populations with antisocial personality, substance misuse is overrepresented (42–44) and can be deleterious to neuropsychological functioning (42, 45, 46). Baliousis (42) examined neuropsychological deficits in offenders with antisocial personality disorder (ASPD) and psychopathy, revealing impairments in executive functions, memory, attention, and visual perception, with previous substance abuse identified as a confounding factor (42).

As noted by Curtis et al. (41), due to the high prevalence of cognitive impairments in people with alcohol and drug use disorders (47, 48) as well as in violent offenders (49), and therefore also violent offenders with substance use disorders, this impairment might limit their ability to benefit from treatment programs. In addition, there is a risk that these impairments could be undetected or underestimated (50). However, these findings have thus far rarely been used to inform practice.

In summary, based on previous studies, neurocognitive deficits have been a constant, but often overlooked factor in offenders. The interaction of substance use and neurocognitive impairments could be a way to predict future criminal behavior. If a relationship between neurocognitive deficits and offending exists, specific treatment options for these individuals could be provided and this would thus have relevant implications for the treating clinicians.

There is a need for further research into neurocognitive impairments in substance abusing offenders. In order to meet this goal, this study assessed selected neurocognitive functions in patients with substance use disorders in a German forensic psychiatric hospital.

The following hypotheses were formulated:

Hypothesis 1: It is hypothesized that on average the patients in the present study will score higher on the sum scale of the BIS-11 compared to the general population in Germany.

Hypothesis 2: It is hypothesized that on average the patients in the present study will perform worse on selected parameters measuring attention and psychomotor speed, executive functions and social and emotional cognition compared to the general population.

Hypothesis 3: A psychometric measurement of impulsivity, as measured by the sum scale of the Barratt Impulsiveness Scale (BIS-11) does not correlate with behavioral measures representing reaction speed and accuracy, sustained attention, decision making, risk behavior, rule detection and flexibility of attention using the Cambridge Neuropsychological Test Automated Battery (CANTAB) in male patients with substance use disorders.

Hypothesis 4: A higher number of entries in the federal central criminal register and earlier age of first offense and first prison sentence correlate with a lower performance on parameters measuring attention and psychomotor speed, executive functions, social and emotional cognition using the Cambridge Neuropsychological Test Automated Battery (CANTAB).

Hypothesis 5: A higher number of entries in the federal central criminal register and earlier age of first offense and first prison sentence predict a lower performance on parameters measuring attention and psychomotor speed, executive functions, social and emotional cognition using the Cambridge Neuropsychological Test Automated Battery (CANTAB).

## 2 Method

### 2.1 Setting

The study was conducted at the Clinic for Forensic Psychiatry in Rostock, Germany, a 103-bedded unit focusing on patients with substance use disorders. Treatment includes cognitive-behavioral therapy (CBT), DBT-F (dialectical-behavioral therapy for forensic settings), R&R (“Reasoning & Rehabilitation”), relapse prevention group for alcohol and substance use, social skills training, as well as occupational and sports therapy. The average length of stay is 2.5 years.

### 2.2 Participants

For this study, male patients (n=109) of the Clinic for Forensic Psychiatry in Rostock were screened with regard to the inclusion-/and exclusion criteria of the study which were:

Inclusion criteria.

- male gender.
- age ≥ 18 years.
- diagnosis of mental and behavioral disorders due to psychoactive substance use (F10-F19) according to ICD 10.
- IQ at least 70 as assessed by the Wechsler Adult Intelligence Scale – Fourth Edition (WAIS-4, 51) or other suitable tests.
- minimum period of abstinence of one month prior to testing.
- capacity to consent.

Exclusion criteria.

- female gender.
- urrent psychosis, severe mood disorder or primary neurological disorders as assessed by the treating physician.
- IQ <70 according to ICD 10 (F70-F79) (52).
- history of traumatic brain injuries leading to hospitalization.
- language difficulties preventing the understanding of the instructions.

Ideally, only participants not being on medication affecting the central nervous system (CNS), including antipsychotics, antidepressants, mood stabilizers, anxiolytics, sedatives, antiepileptics, etc., should be included in the study. However, this was not feasible in practice, so that we chose instead to document which drugs were used. Medication use at the time of the testing was treated as a confounder and dealt with statistically.

Fifty-nine patients who did not meet inclusion criteria, were excluded for the following reasons:

- 19 patients had a psychotic disorder.
- 19 had an IQ test result of <70.
- five patients were still undergoing assessment.
- four had recently used illicit substances.
- three were excluded due to risk to others.
- two patients could not participate due to language difficulties.
- two had already been discharged at the time of testing.
- one patient was under the age of 18.
- one each was diagnosed with: 1. autism, XXY syndrome and had no substance use disorder, 2. dysexecutive syndrome and possible organic personality disorder, 3. amnestic syndrome, 4. fetal alcohol syndrome.

Therefore 50 patients met inclusion criteria, of these 30 consented to participate.

### 2.3 General procedure

Individual appointments were made with the patients after consultation with nursing staff. The measures included the completion of a questionnaire on impulsivity (Barratt Impulsiveness Scale (BIS-11) and the measurement of neuropsychological parameters with the Cambridge Neuropsychological Test Automated Battery (CANTAB) via tablet with a touchscreen(iPad). Seven neuropsychological tests were performed. The first test (Motor Screening Task) only served as a “warm-up” to familiarize participants with the set-up and was not included in the analysis. The order in which the remaining tests were applied was determined by a random procedure. Of the 6 tests, 3 were available with a German voice-over within the test procedure (Reaction Time, Rapid Visual Information Processing, Emotion Recognition Task). The other tests were only available in English (Cambridge Cognition Task, Intra-Extra Dimensional Set Shift, Stockings of Cambridge). Therefore, we translated the English voice-over into German and read this out during specially defined pauses during the test (for further explanations of the tests, see below). The CANTAB tests took approximately 1 hour to complete, extending to a maximum of 1.5 hours with explanations. The tasks were administered in one or two sessions, depending on the patient’s capacity. Participants were allowed to take breaks if necessary, with a regular short break planned after the first four tests.

The BIS-11 was given to the patients on the day of inclusion in the study or on the day of CANTAB testing and subsequently completed. The neuropsychological testing took place between 16/03/2021 and 01/09/2021.

Furthermore, sociodemographic, clinical and criminal data of the patients were collected, including the Symptom Check List-90 Revised scale (SCL-90-R) (53) to assess the presence (and severity) of psychiatric symptoms. The assessment took place at the time of admission of the patients to the clinic (for further information: 54).

### 2.4 Measures

#### 2.4.1 The Barratt impulsiveness scale

This questionnaire has been widely used in criminal populations (55, 56). Furthermore, Haden and Shiva (57) who studied male patients undergoing inpatient forensic treatment, found a correlation between impulsivity tested with the Barratt impulsiveness scale (BIS-11) and drug/alcohol problems measured with the Personality Assessment Inventory (PAI).

The measure consists of 30 items. The response format is forced choice (1= rarely/never, 2=occasionally, 3=often, 4=almost always/always). Total scores therefore can range from 30 to 120. Higher scores indicate more pronounced impulsivity. (58). The American version identified a total of 6 first-order factors (“Attention”, “Motor Impulsivity”, “Self-Control”, “Cognitive Complexity”, “Persistence” and “Cognitive Instability”), which were subsequently combined into 3 second-order factors (“Attention Disinhibition”, “Motor Impulsivity” and “Non-Planning Impulsivity”) (59). In our study, patients were tested with the German version (The questionnaire was kindly provided by the author of the translation.), which had been translated and back-translated by two independent persons (one of whom a native English speaker) (60). For psychometric evaluation, individuals were recruited from various groups: a representative urban population sample from Munich (n=810), psychiatric inpatients with different diagnoses (n=57), individuals with alcohol dependence (n=114), and female patients with borderline personality disorder (n=40) undergoing inpatient treatment (for further information: 60). The translated version did not replicate the original 6-factor structure. However, in the representative population sample, the internal consistency of the BIS-11 total scale was acceptable (α=0.69), and it improved to α=0.74 after excluding item 11. Impulsivity levels varied across the groups. Scores for the alcohol-dependent and suicidal patients were higher than the control group but lower than those for patients with borderline personality disorder. In a separate study, ninety male violent offenders were recruited from a prison (preventive detention, n=36), regular detention (n=31), and a forensic-psychiatric hospital (n=23) in Munich, Germany. Approximately 57.8% of the participants had a substance abuse or dependence diagnosis. BIS-11 total scores were comparable in the three groups (61), indicating the scale’s suitability for measuring impulsivity and its distinction from traits like aggressiveness and emotional instability (60). Confirmatory analysis of the originally suggested factor structure did not adequately represent the data in the sample of the psychometric evaluation in Germany. The BIS-11 sum score, which showed adequate internal consistency in all subgroups, significantly differentiated impulsivity between patients and controls. For this reason, the use of the total scale only is recommended in German-speaking populations and not the subscales suggested in previous studies (60). Based on this, we chose to use only the total scale in our study to measure impulsivity in the recruited patients.

#### 2.4.2 The Cambridge Neuropsychological Test Automated Battery

To measure selected neuropsychological parameters, we used the Cambridge Neuropsychological Test Automated Battery (CANTAB). It was originally developed at the University of Cambridge and comprises highly sensitive, precise and objective measures of cognitive function correlated with neural networks (62). The CANTAB is used to identify neuropsychological impairments and is the most widely used international interactive computer-based neuropsychological test battery. Testing demonstrated high sensitivity to positive and negative pharmacological, genetic and environmental effects in healthy individuals and patient groups across research domains (63).

For this study, tests from three domains within the CANTAB battery were used: Attention and psychomotor speed, executive functions, social and emotional cognition.

##### 2.4.2.1 Attention and psychomotor speed

###### 2.4.2.1.1 Motor screening task

This test assesses participants’ speed of response and the accuracy of pointing (selecting the cross). For this, colored crosses are presented in different locations on the screen, one at a time. The participant has to select the cross on the screen as quickly and accurately as possible. However, as mentioned above, this test only serves as a “warm-up” and was not included in the evaluation. The Administration time is 2 minutes.

###### 2.4.2.1.2 Reaction time

This tests assesses motor and cognitive reaction speed as well as movement time, reaction time, reaction accuracy and impulsivity. The patients’ task is to hold down a button in the lower half of the screen. Circles are displayed above the button. A dot is displayed in each of the circles. Participants are then asked to release the button as quickly as possible and select the circle in which the dot appeared (64). The recommended outcome variables are the Median Reaction Time and Median Movement Time. The Administration time is 3 minutes.

###### 2.4.2.1.3 Rapid visual information processing

This test measures sustained attention. A white field is displayed in the center of the screen where numbers from 2 to 9 appear in a random order at a rate of 100 numbers per minute. Participants are asked to recognize target sequences of numbers (e.g. 2-4-6, 3-5-7, 4-6-8). When this target sequence appears, they are asked to touch the button in the center of the screen as quickly as possible. The level of difficulty varies with one or three target sequences for the patients to pay attention to at the same time (65). The recommended outcome variables are RVP A (A Prime) (a measure of how good the participant is detecting target sequences) and the Median Response Latency. The Administration time is 7 minutes.

##### 2.4.2.2 Executive functions

###### 2.4.2.2.4 Cambridge Gambling Task

In the Cambridge Gambling Task, decision-making and risk behavior are tested. Participants are presented with a series of ten boxes at the top of the screen: Some of the fields are red and others blue. The ratio of red and blue fields varies. A yellow symbol is hidden in one of the boxes. In the lower half of the screen, the boxes “red” and “blue” are displayed. Participants are asked to select the box where they think the symbol might be hidden, either the red or the blue one. Participants start with 100 points. When selecting the box, they are asked to place a wager in the form of points on their choice. The aim is to score as many points as possible. Based on their wager, participants can both gain and lose points. A circle in the middle of the screen shows the current stake, which is gradually increased or decreased depending on the task variant selected. Participants press this button when it shows the proportion of the points they want to bet. Depending on their choice and where the token is actually hidden, these points are added to their total score if they choose correctly or deducted if the yellow token was hidden in the different colored box (66). The recommended outcome variables are the Decision Making Quality (the proportion of all trials where the participant chose the majority box color. It is calculated over all assessed trials), Risk Adjustment (a measure of sensitivity to risk, based on the ability to modify choices in the light of information about the probability of different outcomes and to track the optimal outcome on each trial. The measure is calculated from the average proportion of points participants chose to bet, taking into account the number of colored boxes in the majority) and Delay Aversion (allows for the dissociation between risk taking and impulsivity by determining whether subjects simply just place a bet at the first opportunity). The Administration time is 12-18 minutes.

###### 2.4.2.2.5 Intra-extra dimensional set shift

The IED tests rule detection and reversal. It determines the ability to distinguish visual formations and the flexibility of attention. Two geometric shapes are used in the test: Pink shapes and white lines. In this task, patients are asked to work out a rule based on feedback. After six correct answers, the stimuli and/or the rules changes. Initially, the task includes simple stimuli consisting of only one of the geometric shapes, e.g. two white lines that differ in shape. Later, compound stimuli are used: white lines over the pink shapes. The changes in the rules are initially “intradimensional” (i.e. the pink shapes remain the only relevant shape) and later become “extradimensional” (i.e. white lines become the relevant shape) (67). The recommended outcome variables are the Total Errors (adjusted for the number of completed stages) and the EDS Error Score (number of trials for which the wrong response was given). The Administration time is 7 minutes.

###### 2.4.2.2.6 Stockings of Cambridge

The SOC is a test of spatial planning. Participants are asked to find strategies to solve problems. They are presented with two patterns on the screen. Each of these patterns contains three colored balls. The balls are arranged differently in each display. Participants are asked to move the balls in the lower display and try to copy the pattern shown in the upper display. The number of moves to reach the target should be as low as possible (68). Participants start with a couple of two move problems. Then, the task gets harder and the number of moves needed to copy the top arrangement increases to up to 5 moves needed. The main outcome variables are the Problems solved in Minimum Moves, Average Number of Moves, Initial Thinking Time (Subjects are encouraged to plan their moves before actually starting to solve the problems. This measure therefore provides an indication of the time taken to plan the problem solution, discounting movement time.) and the Subsequent Thinking Time (This measure provides an indication of any time taken by the subject to plan or re-plan the problem solution after they have made their first move taking into account their movement time, and the number of moves made.). The Administration time is 10 minutes.

##### 2.4.2.3 Social and emotional cognition

###### 2.4.2.3.1 Emotion recognition task

This test measures the ability to identify six basic emotions in facial expressions along a continuum of expressiveness. Participants are presented with successive computer-generated images designed from facial features of real people, each depicting a particular emotion. Each face is displayed for 200 ms and immediately masked. The intensities of the emotions in the facial expressions vary. Participants are then shown 6 optional faces that display different emotions (sadness, happiness, fear, anger, disgust or surprise). They are then asked to choose which of the emotions corresponded to the previously shown face (69). The main outcome variables are the Median reaction time and the Total Hits. The Administration time is 6-10 minutes.

##### 2.4.2.4 The symptom check list-90 revised scale

To assess the presence (and severity) of psychiatric symptoms, the Symptom Check List-90 Revised scale (SCL-90-R) (53) was used. The total Global severity index (GSI) is considered a good indicator of general psychological stress (70). A participant has psychological distress of a clinically relevant degree if he or she has a GSI norm score T ≥ 63 and/or T ≥ 63 on at least two subscales (71). Additionally, Gamman and Linaker (72) determined that a raw GSI score of > 1.5 is considered sufficiently sensitive (0.78) and specific (0.87) for screening mental disorders in prisoners (71, 73).

### 2.5 Statistical analyses

All analyses were performed using SPSS Statistics v. 29 (IBM Corp., USA).

To test hypothesis 2, which posits that patients will perform worse on selected parameters measuring attention, psychomotor speed, executive functions, and social and emotional cognition compared to the general population, the CANTAB test results of the study participants were compared with those of healthy individuals from the general population.

For the domain of executive functions, normative data were obtained from the study by Czapla et al. (74). This study tested 71 healthy controls (HC) from the German general population. The sample had a mean age of 46 years (SD = 12.02), with 24% female and 76% male participants. Exclusion criteria for the patient group included current substance abuse or dependence other than nicotine or alcohol, severe somatic, neurological, or psychiatric illness, severe complications of detoxification, pregnancy, lactation, or suicidality.

For psychomotor speed and planning, normative data for the Stockings of Cambridge (SOC) and Reaction Time (RTI) parameters were obtained from the study by Majer et al. (75). This study included 104 control subjects from the general population of Georgia, with a mean age of 43.8 years (SD = 10.9), comprising 27 males and 77 females.

For attention and rule detection, data were acquired using the CANTAB Connect software. By inputting the participants’ birth dates into the system, normative references for each study participant were established. This allowed for the computation of respective comparison values based on the normative data.

The Mann-Whitney U test, a non-parametric test, was employed to assess the differences between the study population and the general population for attention and rule detection tests. This statistical analysis was possible for each data point of a subject in these tests, enabling a comprehensive comparison. However, for the domains of executive functions and psychomotor speed and planning, the Mann-Whitney U test could not be used due to differences in the number of subjects and the heterogeneity of the study designs.

To test Hypothesis 3 and 4, a Partial correlation analysis was used to determine how the CANTAB outcome measures, the Sum Scale of the BIS-11, the number of entries in the federal central criminal register, the age at the first recorded offence and the age of the first prison sentence were related to each other. The dataset was carefully examined to ensure that the assumptions for conducting a valid partial correlation analysis were met. In order to visually check linear relationships, a scatter plot of all variables included in the analysis was created. By calculating Z-scores and visually inspecting boxplots, potential outliers were identified and excluded from the analysis. Outliers exceeding a Z-score of 3 or -3 were considered substantial and were excluded due to their potential influence on the correlation estimates. Regarding missing values, the decision was made not to replace them. Instead, the listwise deletion method was used.

Statistical significance was set at p < 0.05. Correlations were calculated using age, IQ and medication intake at the time of testing as covariates of not interest.

To test Hypothesis 5, a hierarchical elimination regression analysis was used to analyze whether the number of entries in the federal central criminal register, the age at the first recorded offence and/or the age of the first prison sentence of the participants predicted performance on parameters measuring attention and psychomotor speed, executive functions, social and emotional cognition (CANTAB). Therefore, we assessed assumption violations according to standard practice including linearity, homoscedasticity, independence of errors, and normality.

## 3 Results

### 3.1 Patient characteristics

As can be seen in **Table 1**, the mean age of participants was 34 years (SD=8.57; Range= 18-49), the length of stay at the time of testing ranged from one month to 44 months since admission to the clinic (median =9.5, mean = 13.5). Most patients had German nationality (90%), were single/not in a relationship (76.7%) and had a secondary school leaving certificate (nine years) (70%). Half of the participants had completed vocational training.

**Table 1:** Sociodemographic and clinical characteristics.

	N	%	M	SD	Range
Age at assessment (Years)			34	8.6	
Range of Age (Years)					18-49
Nationality					
German	27	90			
other	3	10			
Marital status					
single/not in relationship	23	76.7			
in relationship, but not married	3	10			
married	2	6.7			
divorced/separated	2	6.7			
Education status					
secondary school leaving certificate (after 9 years)	21	70			
secondary school leaving certificate (after 10 years)	6	20			
aborted school career	3	10			
Vocational qualification					
completed	15	50			
none	14	46.7			
information not available in records	1	3.3			
Diagnosis (due to ICD-10)					
Mental and behavioral disorder due to multiple drug use and other psychoactive substances (F19)	15	50			
Mental and behavioral disorder due to use of other stimulants, including caffeine (F15)	7	23.1			
Mental and behavioral disorder due to use of cannabinoids (F12)	6	20			
Mental and behavioral disorder due to use of alcohol (F10)	6	20			
Mental and behavioral disorder due to use of opioids (F11)	3	9.9			
Mental and behavioral disorder due to use of cocaine (F14)	2	6.6			
Mental and behavioral disorder due to use of sedatives or hypnotics (F13)	2	6.6			
No disorders of adult personality and behavior	26	86.7			
Dissocial personality disorder (F60.2)	4	12.2			
Paranoid personality disorder (F60.0)	1	3.3			
Psychotropic Medication					
No psychotropic medication	14	46.7			
Antidepressants	5	16.7			
Opiate substitution treatment	6	20			
Antipsychotics (2^nd^ generation)	2	6.6			
Stimulants and other drugs for the treatment of ADHS	2	6.6			
Hypnotics/Sedatives	2	6.6			
Antiepileptics	2	6.6			
Psychometric Testing					
Intelligence quotient (IQ) (Measured with the WAIS-4)			91.7	10.7	
Range (IQ)					73-116
SCL-90-R (GSI) a			0.5	0.5	
SCL-90-R (GSI T-Score) b			53.3	9.8	
SCL-90-R (PST T-Score) c			52.4	10.0	
SCL-90-R (PSDI T-Score) d			55	8.5	
Legal issues					
Section 64 of the German penal code	28	93.3			
Section 67 g of the German penal code e	1	3.3			
Other (accommodation via administrative assistance)	1	3.3			
Fully criminally responsibility	19	63.3			
Section 21 of the German penal code (partially criminally responsible)	10	33.3			
Section 20 of the German penal code (not criminally responsible)	1	3.3			
Number of Entries in the BZR f			10.7	7.6	
Age at the first recorded offence			18	6.5	
Age at the first recorded offence					14-36
Age of the first prison sentence			22	6.7	
Age at the first prison sentence					15-40
Index offence					
Robbery and extortion	14	46.7			
Narcotics offences	10	33.3			
Offences against physical integrity	9	30			
Theft and embezzlement	6	20			
Offences against life	3	10			
Offences against sexual self-determination	1	3.3			
Other offences	1	3.3			
**Index offence and past offences**	19				
Bodily injury offence		63.3			

a Symptom Check List-90 Revised Scale (Global Severity Index).

b Symptom Check List-90 Revised Scale (Global Severity Index T-Score).

c Symptom Check List-90 Revised (Positive Symptom Total T-Score).

d Symptom Check List-90 Revised (Positive Symptom Distess Index T-Score).

e Revocation of the suspension of a placement (for further information: 6).

f BZR (Bundeszentralregister) - records: Number of entries in the federal central criminal register.

Multiple use of substances was found in 24 of the 30 participants. Six patients were found to use one substance only (three used stimulants including caffeine, one alcohol, one cocaine and one cannabinoids only). Most of the participants (86,7%) were not diagnosed with a personality disorder but four patients had a dissocial personality disorder, one patient had a paranoid and a dissocial personality disorder. The measurement of IQ resulted in a mean value of 91.7 (SD= 10.7; Range= 73-116). At the time of study enrollment, 14 (46.7%) of the patients were not taking any psychotropic medication. 5 (16.7%) of the patients received antidepressants, 6 (20%) received opiate substitution treatment and 2 (6.6%) antipsychotics (2nd generation) (for further information see **Table 1**).

The vast majority of the patients (93%) were admitted under §64 of the German penal code. Nineteen patients were considered fully criminal responsible at the time of the index offence, ten of reduced criminal responsibility and one was criminally irresponsible. The mean number of entries in the federal criminal register was 10.7 (SD= 7.6). The age at first entry was on average 18.8 (SD=6.5; Range=14-36). The age at the first prison sentence was on average 22.1 (SD=6.7; Range=15-40). The index offence of 46.7% of the patients was robbery and extortion, followed by narcotics offences with 33.3%. 63.3% of the patients had a record of bodily injury offences (as an index offence as well as in the past).

For the SCL-90, **Table 1** presents the descriptive statistics for a sample of 29 participants. For one participant, no SCL-90 was collected. The Global Severity Index (GSI) scores ranged from a minimum of 0.04 to a maximum of 2.5, with a mean of 0.5 (SD = 0.5), indicating relatively low levels of psychological distress. The T-Score for the GSI ranged from 36 to 77, with a mean of 53.3 (SD = 9.8), suggesting that participants’ scores fell within the average range of the severity of psychiatric symptoms measured with the SC-90-R.

### 3.2 CANTAB test results

**Table 2** provides a list of all CANTAB parameters collected and the Barratt Impulsiveness Scale (BIS-11) results. The mean and standard deviation of each parameter are presented, along The main outcomes are highlighted.

**Table 2:** Reference values of the CANTAB, BIS-11 compared with the patient population.

Neurocognitive domain	Outcome Measures	Measure Name	Unit	Sense*	Range	Standard Score	Min	Max	Mean	SD
**Attention and Psychomotor Speed**	**Reaction Time (RTI)**									
	Simple Median Reaction Time	RTISMDRT	Continuous/ ms	-ve	100-5100	340,2	229,0	362,0	305,2	32,5
	Simple Median Movement Time	RTISMDMT	Continuous/ms	-ve	100-5100	445,3	134,0	378,0	219,3	53,7
	Median Five- Choice Reaction Time	RTIFMDRT	Continuous/ms	-ve	100-5100	369,4	287,0	466,0	352,4	37,3
	Median Five- Choice Movement Time	RTIFMDMT	Continuous/ms	-ve	100-5100	434,4	145,0	388,5	254,2	58,2
	**Rapid Visual Information Processing (RVP)**									
	RVP A´	RVPA	Discrete	+ve	0-1	0,9	0,8	1,0	0,9	0,0
	Median Response Latency	RVPMDL	Continuous/ms	-ve	100-1900	411,7	339,0	708,0	436,8	78,5
**Executive Functions**	**Cambridge Gambling Task (CGT)**									
	Decision Making Quality Total Merged	CGTDMQMT	Discrete	+ve	0-1	0,9	0,4	1,0	0,9	0,1
	Risk Adjustment Merged	CGTRAJMT	Discrete	cx	(-)5.4-5.4	1,3	-1,2	2,6	0,9	1,0
	Delay Aversion Total	CGTDAVT	Discrete	cx	(-)0.9-0.9	0,3	-0,1	0,8	0,2	0,2
	**Intra-Extra Dimensional Set Shift (IED)**									
	Total Errors (Adjusted)	IEDYERTA	Discrete	-ve	0-402	24,8	8,0	182,0	29,1	35,7
	EDS Error Scores	IEDEEDS	Discrete	-ve	0-50	9,4	0,0	30,0	7,4	6,6
	**Stockings of Cambridge (SOC)**									
	Problems Solved in Minimum Moves Total (All Moves)	SOCPSMMT	Discrete	+ve	0-12	8,7	3,0	12,0	8,1	2,6
	Mean Moves (5 Moves)	SOCMNM5	Discrete	-ve	5 to 12	6,6	5,0	12,0	7,2	1,8
	Initial Thinking Time Median (5 Moves)	SOCITMD5	Continuous/ms	-ve	0-Unlimited	13200,00	1798,0	34406,0	8266,4	6198,4
	Subsequent Thinking Time Median (5 Moves)	SOCSTMD5	Continuous/ms	-ve	0-Unlimited	1300,00	0,0	7130,6	1134,1	1892,9
**Social and Emotional Cognition**	**Emotion Recognition Task (ERT)**									
	Median Reaction Time	ERTRT	Continuous/ms	-ve	0-Unlimited		1374,0	6667,0	3644,2	1609,8
	Total Hits	ERTTH	Discrete	+ve	0-90		37,0	67,0	53,3	7,6
	Unbiased Hit Rate Sadness	ERTUHRS	Discrete	+ve	0-1		0,1	0,6	0,4	0,2
	Unbiased Hit Rate Fear	ERTUHRF	Discrete	+ve	0-1		0,0	0,5	0,2	0,1
	Unbiased Hit Rate Anger	ERTUHRA	Discrete	+ve	0-1		0,1	0,7	0,4	0,1
	Unbiased Hit Rate Surprise	ERTUHRSU	Discrete	+ve	0-1		0,2	0,8	0,4	0,1
	Unbiased Hit Rate Disgust	ERTUHRD	Discrete	+ve	0-1		0,1	0,8	0,4	0,2
	**Barratt Impulsiveness Scale (BIS-11)**									
	Sum Scale of the BIS-11		Discrete	+ve	30-120	57,2	44,0	86,0	62,5	10,0

*+ve = Higher score indicates better performance.

-ve = Lower score indicates better performance.

cx= Measure sense is complex.

Neuropsychological assessments across various domains revealed specific performance measures. Notably, in the domain of psychomotor speed, participants demonstrated moderate mean scores across parameters such as Simple Median Reaction Time, Simple Median Movement Time, Median Five-Choice Reaction Time, and Median Five-Choice Movement Time. Performance on the Rapid Visual Information Processing (RVP), as a measure of attention, indicated relatively high mean RVP A scores, suggesting good performance, while the Median Response Latency showed moderate scores.

Executive functions, as measured by the Cambridge Gambling Task (CGT), revealed generally high mean scores across Decision Making Quality Total Merged (The proportion of all trials where the participant chose the majority box color calculated over all assessed trials). In problem-solving tasks, participants showed moderate to high mean scores across Problems Solved in Minimum Moves Total and moderate Mean Scores in Mean Moves.

Social and emotional cognition assessments demonstrated variable performance across parameters, with generally moderate mean scores. These results underscore the diverse neuropsychological profile of the participants, highlighting strengths in certain cognitive domains and areas for potential impairment in others.

### 3.3 BIS-11 test results

The Sum Scale of the BIS-11 was measured with a range from 30 to 120. The mean score war 62.5 (SD=10.0).

### 3.4 Comparison of the BIS-11 data between study participants and the general population

To test Hypothesis 1, our test results were compared with the general population. The latter sample consisted of individuals recruited from Munich (n=810, 369 males, 441 females, mean age: 47.28 years, SD=14.9) (60). Compared to the German general population (60), who scored a mean of 57.2 on the Sum scale of the BIS-11, the participants of the present study scored higher (mean=62.5, Range=30-120, SD=10.0) (see **Table 2**).

### 3.5 Comparison of the neuropsychological parameters attention, psychomotor speed and executive functions (CANTAB) between study participants and the general population

In Hypothesis 2, It is hypothesized that patients will perform worse on selected parameters measuring attention and psychomotor speed, executive functions and social and emotional cognition compared to the general population.

#### 3.5.1 Executive functions

The study by Czapla et al. (74) provided normative data for the Cambridge Gambling Task (CGT) and Intra/Extra-Dimensional Set Shift (IED). In this study, 71 healthy controls from the German general population were tested. The results indicated that the German general population scored comparable to the patient group on the CGT. However, our patient group performed slightly worse than the general population on the IEDYERTA (Total Errors, adjusted for the number of completed stages), with mean scores of 29.1 for participants and 24.8 for the general population. Conversely, the patient group performed better than the general population on the IEDEEDS (number of trials for which the wrong response was given), with mean scores of 7.4 for participants and 9.4 for the general population.

#### 3.5.2 Attention and rule detection

For the Rapid Visual Processing (RVP A´) test, which measures attention and the ability to detect target sequences, the study participants performed significantly better than the general population. The mean score for participants was 0.9, compared to -0.24 for the general population. The Mann-Whitney U test results indicated significant differences between the two groups (Mann-Whitney U = 0.000; Z = -6.596; p < 0.001, two-tailed), with an effect size of r = -0.86, suggesting a very strong effect.

For the Intra/Extra-Dimensional Set Shift (IEDYERTA), the general population performed significantly better than the study population, with mean scores of 0.25 for the general population and 29.1 for the participants. The Mann-Whitney U test results demonstrated significant differences (Mann-Whitney U = 0.000; Z = -6.656; p < 0.001, two-tailed), with an effect size of r = -0.86, indicating substantial differences in cognitive flexibility and rule detection skills.

The results for the IEDEEDS showed that the general population outperformed the study group, with mean scores of 0.27 for the general population and 7.4 for the participants. The Mann-Whitney U test results indicated significant performance differences (Mann-Whitney U = 49.000; Z = -5.626; p < 0.001, two-tailed), with an effect size of r = -0.752, highlighting a strong difference in error rates on the IED EDS task.

#### 3.5.3 Psychomotor speed and planning

In the study by Majer et al. (75), which included 104 control subjects with a mean age of 43.8 years (SD = 10.9), the patient population in our study showed lower performance on the Stockings of Cambridge (SOCPSMMT) compared to normative data, with mean scores of 8.1 for the patient population and 8.7 for the general population. The general population had both a longer Initial Thinking Time Median (SOCITMD5), with mean scores of 13200.0 ms compared to 8266.4 ms for the patient population, and a longer Subsequent Thinking Time (SOCSTMD5), with mean scores of 1300.0 ms compared to 1134.2 ms for the patient population.

Regarding the Reaction Time (RTI) parameters, the general population had slightly longer reaction times and significantly longer movement times compared to the patient population. The mean reaction times were 340.2 ms for the general population and 305.2 ms for the patient population (RTISMDRT), and 369.4 ms for the general population and 352.4 ms for the patient population (RTIFMDRT). Movement times were 445.3 ms for the general population compared to 219.3 ms for the patient population (RTISMDMT), and 369.4 ms for the general population compared to 352.4 ms for the patient population (RTIFMDM).

### 3.6 Correlation between the neuropsychological parameters attention, psychomotor speed and executive functions (CANTAB) and impulsiveness (BIS-11)

To test hypothesis 3, which proposed that the BIS-11 does not correlate with behavioral measures such as reaction speed and accuracy, sustained attention, decision making, risk behavior, rule detection and flexibility of attention using CANTAB, we conducted a partial correlation analysis controlling for the age of the participants, their IQ and medication intake at the time of testing. The partial Pearson correlation analysis revealed that most of the CANTAB outcome measures didn´t correlate with the Sum Scale of the BIS-11 (see **Table 3**).

**Table 3:** Partial Correlation between the Neuropsychological parameters Attention and Psychomotor Speed and Executive Functions (CANTAB) and Impulsiveness (BIS-11).

Domain	Measures	
**Impulsiveness a** **Attention and Psychomotor Speed a, c** **Executive Functions a, c** **Impuliveness b** **Attention and Psychomotor** **Speed b, d** **Executive Functions b, d**		BIS 11
	BIS 11	1.00
	RTISMDRT	-0.35
	RTISMDMT	-0.32
	RTIFMDRT	-0.02
	RTIFMDMT	-0.29
	RVP A´	0.12
	RVPMDL	-0.17
	CGTDMQMT	-0.15
	CGTRAJMT	-0.45*
	CGTDAVT	0.11
	IEDYERTA	-0.04
	IEDEEDS	0.21
	Age	-0.18
	IQ	-0.22
	Medication intake	0.46*
	BIS-11	1.00
	RTISMDRT	-0.23
	RTISMDMT	-0.34
	RTIFMDRT	0.17
	RTIFMDMT	-0.25
	RVP A´	0.18
	RVPMDL	-0.01
	CGTDMQMT	-0.11
	CGTRAJMT	-0.55*
	CGTDAVT	0.18
	IEDYERTA	-0.11
	IEDEEDS	0.09

BIS-11, Barrat Impulsiveness Scale; RTISMDRT, Reaction Time Simple Median Reaction Time; RTISMDMT, Reaction Time Simple Median Movement Time;RTIFMRT, Reaction Time Median-Five Choice Reaction Time.

RTIFMDMT, Reaction Time Median Five-Choice Movement Time; RVPMDL, Rapid Visual Information Processing Median Response Latency; CGTDMQMT Cambridge Gambling Task Decision Making Quality Total Merged; CGTRAJMT, Cambridge Gambling Task Risk Adjustment Merged; CGTDAVT, Cambridge Gambling Task Delay Aversion Total; IEDYERTA Intra-Extra Dimensional Set Shift Total Errors (Adjusted); IEDEEDS, Intra-Extra Dimensional Set Shift EDS Error Scores; Age, Age of the patient; Medication intake, Medication intake at the time of testing.

*p<0.05.

a Cells contain zero-order (Pearson) correlations. b controlling for Age of the participant & IQ of the patient & Medication intake at the time of testing. c df=21. d df=18.

In case of a zero-order correlation, without controlling for possible confounders, only one outcome measuring Executive functions correlated with the BIS-11. The Result of the Sum scale of the BIS-11 and the CGTJATM (a measure of the patient´s sensitivity to risk, based on the ability to modify choices in the light of information about the probability of different outcomes and to track the optimal outcome on each trial) (r=-0.449, p=0.031) correlated negatively meaning more impulsive patients were less sensitivity to risk and vice versa. The proportion of common variance was 20.2% (r2 = 0.202). The correlation coefficient was -0.449, which corresponds to a moderate effect according to Cohen (76). When controlling for possible confounders, the Result of the Sum scale of the BIS-11 and the CGTJATM (r=-0.551, p=0.012) correlated significantly. The proportion of common variance was 30.4% (r2 = 0.304). The correlation coefficient was -0.551, which corresponds to a strong effect according to Cohen (76).

### 3.7 Correlation between criminal history and neuropsychological parameters

In hypothesis 4, it was supposed, that a higher number of entries in the federal central criminal register and earlier age of first offense and first prison sentence would correlate with a lower performance on parameters measuring attention and psychomotor speed, executive functions, social and emotional cognition using the CANTAB. To test this hypothesis, we conducted a Partial correlation analysis controlling for the age of the participants, their IQ and medication intake at the time of testing (see **Table 4**).

**Table 4:** Partial Correlation between criminal Data and Neuropsychological parameters, conducted by the CANTAB.

Domain		Measures		
		BZR records	Age first BZR record	Age first prison sentence
**Criminological data a, c** **Attention and Psychomotor speed a, c** **Executive Functions a, c** **Emotional and Social cognition a, c** **Criminological data b, d** **Attention and Psychomotor speed b, d** **Executive Functions b, d** **Emotional and Social cognition b, d**	BZR records	1.00		
	Age first BZR record	-0.69**	1.00	
	Age first prison sentence	-0.65**	0.90	1.00
	RTISMDRT	-0.24	0.24	0.10
	RTISMDMT	-0.07	0.21	0.20
	RTIFMDRT	-0.24	0.15	0.10
	RTIFMDMT	-0.16	0.20	0.15
	RVP A´	0.66**	-0.51*	-0.43
	RVPMDL	-0.14	0.06	0.06
	CGTDMQMT	0.28	-0.21	-0.28
	CGTRAJMT	0.16	0.06	0.07
	CGTDAVT	-0.30	0.39	0.39
	IEDYERTA	-0.05	0.21	0.09
	IEDEEDS	0.08	-0.08	-0.18
	SOCPSMMT	0.38	-0.40	-0.23
	SOCMNM5	-0.31	0.51*	0.39
	SOCITMD5	0.49*	-0.44	-0.32
	SOCSTMD5	-0.20	0.52*	0.48*
	ERTRT	-0.15	0.30	0.17
	ERTTH	-0.10	-0.06	-0.18
	ERTUHRS	-0.04	-0.02	-0.10
	ERTUHRF	-0.22	0.10	-0.00
	ERTUHRA	-0.15	-0.15	-0.24
	ERTUHRSU	-0.03	-0.13	-0.18
	ERTUHRD	-0.20	0.15	0.14
	Age	0.29	0.03	0.06
	IQ	0.46*	-0.15	-0.01
	Medication intake	0.56*	-0.58	-0.52*
	BZR records	1.00		
	Age first BZR record	-0.56*	1.00	
	Age first prison sentence	-0.61*	0.87	1.00
	RTISMDRT	0.03	-0.06	-0.16
	RTISMDMT	-0.06	0.18	0.17
	RTIFMDRT	-0.17	0.02	-0.01
	RTIFMDMT	-0.01	0.08	0.03
	RVP A´	0.53*	-0.38	-0.42
	RVPMDL	-0.06	-0.12	-0.10
	CGTDMQMT	0.14	-0.13	-0.29
	CGTRAJMT	0.11	0.13	0.09
	CGTDAVT	-0.09	0.28	0.35
	IEDYERTA	0.25	0.11	0.06
	IEDEEDS	0.16	-0.07	-0.13
	SOCPSMMT	0.15	-0.28	-0.15
	SOCMNM5	-0.18	0.48	0.38
	SOCITMD5	0.48	-0.34	-0.18
	SOCSTMD5	-0.07	0.50*	0.50*
	ERTRT	0.12	0.18	0.07
	ERTTH	0.22	-0.21	-0.33
	ERTUHRS	0.27	-0.07	-0.16
	ERTUHRF	-0.05	0.13	-0.01
	ERTUHRA	0.10	-0.25	-0.31
	ERTUHRSU	0.02	-0.17	-0.27
	ERTUHRD	0.12	-0.24	-0.16

BZR records, Bundeszentralregister (number of entries in the federal central criminal register), RTISMDRT, Reaction Time Simple Median Reaction Time; RTISMDMT, Reaction Time Simple Median Movement Time;RTIFMRT, Reaction Time Median-Five Choice Reaction Time.

RTIFMDMT, Reaction Time Median Five-Choice Movement Time; RVPMDL, Rapid Visual Information Processing Median Response Latency; CGTDMQMT Cambridge Gambling Task Decision Making Quality Total Merged; CGTRAJMT, Cambridge Gambling Task Risk Adjustment Merged; CGTDAVT, Cambridge Gambling Task Delay Aversion Total; IEDYERTA Intra-Extra Dimensional Set Shift Total Errors (Adjusted); IEDEEDS, Intra-Extra Dimensional Set Shift EDS Error Scores; SOCPSMMT, Stockings of Cambridge Problems Solved in Minimum Moves Total (All Moves); SOCMNM5, Stockings of Cambridge Mean Moves (5 Moves).

SOCITMD5, Stockings of Cambridge Initial Thinkings Time Median (5 Moves); SOCSTMD5, Stockings of Cambridge Subsequent Thinking Time (5 Moves); ERTRT, Emotion Recognition Task Median Reaction Time; ERTTH, Emotion Recognition Task Total Hits; ERTUHRS, Emotion Recognition Task Unbiased Hit Rate Sadness; ERTUHRF, Emotion Recognition Task Unbiased Hit Rate Fear;.

ERTUHRA, Emotion Recognition Task Unbiased Hit Rate Anger; ERTUHRSU, Emotion Recognition Task Unbiased Hit Rate Surprise; ERTUHRD, Emotion Recognition Task Unbiased Hit Rate Disgust.

**p<0.01, *p<0.05.

a Cells contain zero-order (Pearson) correlations.b controlling for Age of the participant & IQ of the patient & Medication intake at the time of testing. c df=17. d df=14.

In case of zero-order correlation, the BZR-records of the participants (r=0.662, p=0.002, r2 = 0.439) and the age of the first BZR- record (r=-0.509, p=0.026, r2 = 0.259) correlated significantly with the RVP A` (a measure of how good the participant is detecting target sequences in the domain of attention) showing a strong effect according to Cohen (76). There was a positive linear correlation between both measures. Furthermore, the SOC Initial Thinking Time Median (5 Moves), as an indicator of the time taken to plan the problem solution, correlated with the BZR-records (r=0.487, p=0.034, r2 = 0.237) with a moderate effect. There was a positive linear correlation between the age of the first BZR-record and the SOC Mean Moves (5 Moves) (r=0.507, p=0.027, r2 = 0.257) and the SOC Subsequent Thinking Time Median (5 Moves) (r=0.518, p=0.023, r2 = 0.268) with a strong effect. The SOC Subsequent Thinking Time Median (5 Moves), providing an indication of the time taken by the subject to plan or re-plan the problem solution after they have made their first move, also correlated with the age of the first prison sentence (r=0.478, p=0.038, r2 = 0.228) with a moderate effect.

When controlling for the age of the participants, IQ and medication intake at the time of testing, there was a positive linear correlation between the RVP A´ (r=0.527, p=0.036, r2 = 0.278) and the BZR- records. The SOC Subsequent Thinking Time Median (5 Moves) correlated with the age of the first BZR-record (r=0.500, p=0.049, r2 = 0.250) and the age of the first prison sentence (r=0.504, p=0.046, r2 = 0.254) showing a strong effect according to Cohen (76).

### 3.8 Criminal history data as predictor for CANTAB test results

In hypothesis 5, it was supposed, that a higher number of entries in the federal central criminal register and earlier age of first offense and first prison sentence would predict a lower performance on parameters measuring attention and psychomotor speed, executive functions, social and emotional cognition using the CANTAB. To evaluate Hypothesis 5, a hierarchical linear regression analysis was conducted. Model 1 incorporated the number of BZR (Federal Central Criminal Register) records as the primary predictor. Subsequently, Model 2 extended the analysis by including the age at first BZR-record entry and the age at first prison sentence as independent variables. Model 3 further adjusted for potential confounders, namely the participant’s age, IQ, and medication intake status. This analytical framework was applied across 22 dependent variables, specifically performance metrics derived from the CANTAB assessments described above.

Prior to hypothesis testing, the fulfillment of regression analysis prerequisites was verified. The assumption of linearity was substantiated through the examination of partial regression plots. Homoscedasticity was assessed by plotting standardized residuals (ZRESID) against standardized predicted values (ZPRED), revealing no evident deviations from this assumption. The Durbin-Watson statistic, yielding a value proximal to 2, indicated the satisfactory adherence to the independence of errors criterion for the models yielding statistically significant results. Additionally, the investigation into multicollinearity confirmed its absence, as evidenced by tolerance values exceeding 0.1 and variance inflation factors (VIFs) remaining below the threshold of 10.

The hierarchical regression analysis revealed that none of the independent variables produced significant results for RTI, CGT, IED and ERT.

The analysis aimed at evaluating Hypothesis 5 elucidated the predictive accuracy for RVPA´ (a measure of how good the participant is detecting target sequences) performance as one of the tests measuring sustained attention through three sequential models, incorporating progressively comprehensive predictor sets. Model 1, focusing solely on BZR records, explained a modest 9.9% of variance in RVPA´ (R² = .099, adjusted R² = .065) but did not reach statistical significance (F(1, 27) = 2.954, p = .097). The expansion in Model 2 to include age at the first BZR record and age at the first prison sentence slightly improved explanatory power (R² = .186, adjusted R² = .088), yet the enhancements did not statistically augment the model’s predictiveness (F(2, 25) = 1.343, p = .279). The substantial advancement was observed in Model 3 with the addition of participant’s age, IQ, and medication intake as possible confounders, markedly increasing the R² to.576 and adjusted R² to.460, reflecting a significant improvement in model performance (F(3, 22) = 6.744, p = .002). Within this model, participant’s age (B = -.004, Beta = -.630, t = -3.279, p = .003) and IQ (B = .002, Beta = .516, t = 3.349, p = .003) emerged as significant predictors of RVP A´ outcomes. Medication intake, despite a negative association (B = -.029, Beta = -.292), did not achieve statistical significance (t = -1.588, p = .127). These findings emphasize the nuanced role that individual differences—particularly age and IQ—play over mere criminal history records in determining cognitive performance as measured by RVP A´ (see **Table 5**).

**Table 5:** Multiple Regression Analysis with RVPA as dependent variable.

	Model 1		Model 2		Model 3	
	B	p	B	p	B	p
(Constant)	0,870	0.000***	0,928	0.000***	0,799	0.000***
BZR records of the participant	0,002	0,097	0,001	0,559	0,003	0,077
Age of the first BZR record			-0,003	0,276	-0,002	0,417
Age of the first prison sentence			0,001	0,774	0,001	0,669
Age of the participant					-0,004	0,003**
IQ of the patient					0,002	0,003**
Medication intake at the time of testing					-0,029	0,127

Model 1 F=2.95; adj r2=-0.065. Model 2 F=1.91; adj r2 = 0.088. Model 3 F=4.98; adj r2 = 0.460.

*** p<0.001, ** p<0.01.

BZR records, Bundeszentralregister (number of entries in the federal central criminal register).

Assessing the SOC as a test measuring planning behavior of the participants, the SOC Mean Moves (5 Moves) achieved significant results. In the analysis, Model 1’s inclusion of BZR records as a predictor revealed minimal variance explanation (R² = .017, adjusted R² = -.019) without statistical significance (F = .473, p = .497). Model 2 added age at the first BZR record and prison sentence, significantly improving explanatory power (R² = .285, adjusted R² = .200; F = 3.328, p = .036), with age at the first BZR record emerging as a notable predictor (Beta = .762, p = .050) (see **Table 6**). Model 3 incorporated additional confounders including IQ, medication intake, and participant age, slightly increasing variance explained (R² = .304, adjusted R² = .114) but without enhancing predictive significance (F = 1.599, p = .195). The Durbin-Watson statistic of nearly 2 indicated satisfactory independence of residuals. This analysis demonstrates that while age at the first BZR record provided modest predictive value, the overall model had limited explanatory power for SOC Mean Moves (5 Moves), reflecting the complexity of predicting cognitive task performance.

**Table 6:** Multiple Regression as SOC Mean Moves (5Moves) as dependent variable.

	Model 1		Model 2		Model 3	
	B	p	B	p	B	p
(Constant)	7,507	0.000***	3,798	0,014*	5,582	0,101
BZR records of the participant	-0,031	0,497	0,046	0,335	0,067	0,326
Age of the first BZR record			0,209	0,0498*	0,209	0,080
Age of the first			-0,048	0,609	-0,032	0,755
Age of the participant					-0,015	0,779
IQ of the patient					-0,021	0,532
Medication intake at the time of testing					0,087	0,917

Model 1 F=0.47; adj r2=-0.019. Model 2 F=3.33; adj r2 = 0.200. Model 3 F=1.599; adj r2 = 0.114.

*** p<0.001, *p<0.05.

BZR records, Bundeszentralregister (number of entries in the federal central criminal register).

Of the SOC, the SOC Initial Thinking Time Median (5 moves) as an indicator of the time taken to plan the problem solution, reached statistical significance. Targeting this dependent variable, the sequential introduction of variables across three models illuminated their varying degrees of impact: Only Model 1 including BZR records of the participant as the only predictor led to a significant explanation of variance (R² = .230, adjusted R² = .200, F(1, 26) = 7.762, p = .010). The BZR records demonstrated a notable effect (B = 234.711, Beta = .479, t = 2.786, p = .010), signifying a substantial contribution to the initial thinking time. Among adding other predictors or confounders, none of the other Models reached a level of statistical significance that would underscore their individual predictive importance for SOC Initial Thinking Time Median (see **Table 7**).

**Table 7:** Multiple Regression with SOC Intial Thinking Time Median (5 Moves) as dependent variable.

	Model 1		Model 2		Model 3	
	B	p	B	p	B	p
(Constant)	4683,537	0,000***	4786,164	0,141	16127,227	0,023*
BZR records of the participant	234,711	0,00983**	206,704	0,053	366,072	0,013*
Age of the first BZR record			-220,418	0,330	-295,844	0,212
Age of the first prison sentence			197,119	0,336	302,359	0,150
Age of the participant					-109,245	0,307
IQ of the patient					-103,115	0,135
Medication intake at the time of testing					-1728,615	0,317

Model 1 F=7.762; adj r2=-0.200. Model 2 F=2.847; adj r2 = 0.170. Model 3 F=2.222; adj r2 = 0.214.

*** p<0.001, ** p<0.01,*p<0.05.

BZR records, Bundeszentralregister (number of entries in the federal central criminal register).

In the analysis focusing on SOC Subsequent Thinking Time Median (5 Moves) as an indicator of any time taken by the subject to plan or re-plan the problem solution after they have made their first move, significant advancements were observed with Model 2, where the inclusion of age at the first BZR record and the first prison sentence alongside BZR records significantly improved the model’s explanatory power (R² = .334, adjusted R² = .251, F(3, 24) = 4.020, p = .019). This model, marked by a significant shift in the constant to -2950.678 (p = .032), suggested that the collective addition of these variables offered a meaningful enhancement in predicting subsequent thinking times. Despite further variable additions in Model 3—including IQ, medication intake, and participant age—which slightly raised the explained variance to R² = .432 (adjusted R² = .270), the changes did not significantly increase the predictive accuracy of individual factors. This analysis underscores that Model 2 provided a substantial increase in understanding the SOC Subsequent Thinking Time Median, highlighting the significance of considering both criminal history and early life behaviors in predicting cognitive task performance (see **Table 8**). However, the complexity of adding more personal and clinical factors in Model 3 did not yield additional significant predictive benefits, emphasizing the nuanced challenge of identifying key determinants within such cognitive assessments.

**Table 8:** Multiple Regression with SOC Subsequent Thinking Time Median (5 Moves) as dependet variable.

	Model 1		Model 2		Model 3	
(Constant)	1110,501	0,047*	-2950,678	0,032*	-792,767	0,762
BZR records of the participant	-13,506	0,736	64,137	0,130	45,924	0,404
Age of the first BZR record			150,059	0,094	107,942	0,247
Age of the first prison sentence			16,081	0,839	21,293	0,794
Age of the participant					59,700	0,169
IQ of the patient					-35,600	0,202
Medication intake at the time of testing					-128,893	0,847

Model 1 F=0.116; adj r2=-0.034. Model 2 F=4.020; adj r2 = 0.251. Model 3 F=2.663; adj r2 = 0.270.

*p<0.05.

BZR records, Bundeszentralregister (number of entries in the federal central criminal register).

## 4 Discussion

In our study conducted at the Clinic for Forensic Psychiatry in Rostock, Germany, we aimed to elucidate the neuropsychological profile and impulsivity levels in male patients with substance use disorders and their relationship with criminal history. This comprehensive investigation led us to test five hypotheses regarding impulsivity, cognitive performance, and their correlations with criminal data, leveraging the Cambridge Neuropsychological Test Automated Battery (CANTAB) and the Barratt Impulsiveness Scale (BIS-11). The results elucidate several key findings that contribute to our understanding of this population’s neuropsychological and behavioral profiles, inviting further discussion on their implications for treatment and rehabilitation.

Regarding Hypothesis 1, our findings showed that patients exhibited higher impulsivity levels on the BIS-11 compared to the general German population, reflecting heightened impulsivity among this forensic population. This aligns with existing literature on substance use and impulsivity. For example, Huddy et al. (31) demonstrated that impulsivity is a significant factor in adverse outcomes such as substance use and problem gambling, with negative outcomes associated with both self-report and behavioral measures of impulsivity. de Tribolet-Hardy et al. (61) in a study conducted by the Department for Forensic Psychiatry at the University Hospital of Psychiatry in Zurich with 90 male violent offenders were showed scores of between 60.7 (SD=9.5) and 61.7 (SD=12.3) in different detention settings.

However, this raises critical questions about the specificity and sensitivity of the BIS-11 in differentiating between impulsivity as a trait versus state characteristic in forensic populations. The scale’s ability to capture the nuanced expressions of impulsivity, potentially exacerbated by substance use, within a forensic context warrants further scrutiny. This is particularly pertinent when considering the role of impulsivity in criminal behavior, where impulsivity may not only be a predisposing trait but also a consequence of the forensic environment or substance withdrawal.

Hypothesis 2 was partially supported; patients displayed mixed outcomes in neuropsychological domains, performing comparably or slightly worse than the general population in specific tasks, indicating varied cognitive impairments. These findings highlight the heterogeneity within the forensic psychiatric population regarding cognitive functioning, with certain individuals exhibiting comparable or slightly diminished capacities in specific domains. For example, Yücel et al. (24) reviewed relevant studies and suggested that chronic abuse of a wide range of addictive substances can impair neuropsychological functioning, affecting areas such as attention, learning, memory, visuospatial abilities, and executive functions. This aligns with our findings of varied cognitive impairments. The cognitive performance underscores the potential influence of factors such as substance type, duration of use, and comorbid psychiatric conditions, which could not be fully disentangled in this study. Moreover, the direct comparison with normative data from the general population may not adequately account for the socio-demographic and clinical complexities inherent to forensic psychiatric populations, including the impact of institutionalization on cognitive functions.

Hypothesis 3 revealed a weak correlation between impulsivity measures and cognitive performance, which is also found in other studies, suggesting that impulsivity as measured by the BIS-11 does not directly predict neuropsychological task outcomes within this patient cohort. For example, Dolan and Fullam, (34) examined the relationship between psychometric and behavioral measures of impulsivity in a personality disordered population and found weak correlations between these measures, indicating that they may assess different constructs. This finding prompts a re-evaluation of the theoretical frameworks used to understand impulsivity and cognition’s interplay in criminal behavior.

Hypothesis 4, proposing a correlation between criminal history and cognitive performance, was partially supported. Patients’ criminal records and age at first offense were linked with performances on sustained attention and planning behavior, compelling insights into the cognitive correlates of criminal behavior. However, this association also invites skepticism regarding the causality direction and the extent to which cognitive deficits precede or result from criminal engagement. For instance, Ogilvie et al. (35) suggested that neuropsychological deficits in offenders with a history of substance use might increase the risk of engaging in criminal behavior, highlighting the complex interplay between cognitive impairments and criminal activity. The role of external factors, including socioeconomic status, educational background, and access to mental health services, in shaping both criminal behavior and cognitive development remains to be fully explored.

Hypothesis 5 was validated through hierarchical linear regression analyses, particularly for the RVP A´ and SOC tasks, highlighting the predictive power of criminal history over cognitive performance in tasks measuring sustained attention and planning behavior. For RVP A´, age and IQ significantly enhanced predictive accuracy, underscoring individual differences. SOC Mean Moves revealed age at the first criminal record as a significant predictor, although overall model explanatory power was limited. SOC Initial Thinking Time was significantly predicted by criminal history alone, with no improvement with additional personal factors. SOC Subsequent Thinking Time showed significant improvement with age-related criminal history, but additional factors did not provide significant benefits. This aligns with findings by Querengässer and Baur, (22), who identified that static historical variables, particularly criminal history, were significant predictors of treatment outcomes in forensic populations. These findings underscore the importance of considering criminal history in understanding cognitive performance and planning behavior.

The findings of this study extend beyond the original hypotheses by revealing a complex relationship between decision-making, impulsivity, and criminal history data in individuals with substance use disorders. While decision-making quality as an important factor and measured by the CGT (mean = 0.9, SD = 0.1), was relatively high, it was not significantly associated with the BIS-11 or criminal history, such as the age of first criminal record or prison sentence. This aligns with Dolan and Fullam, (34), who also found weak correlations between psychometric impulsivity measures and behavioral tasks. Although decision-making quality did not directly correlate with criminal history, previous studies, including Ogilvie et al. (35) and Curtis et al. (41), suggest that such cognitive deficits can increase the likelihood of criminal behavior. These findings underscore the importance of decision-making as a critical factor in real-world behavior. Tailored interventions focusing on real-world decision-making and impulse control could improve treatment outcomes.

The study’s methodology and analytical strategy are not without limitations. We did not collect data from a healthy control group. Comparison data came from various studies with heterogeneous designs in different settings and countries. Comparison data could not be found for all tasks. Furthermore, not all patients were at a similar stage of treatment; some had not been in hospital for long, while others were about to be discharged. It was also not possible to include the type and frequency of medication taken as a confounder in the analysis, which may have affected the results.

Given the influence of external factors such as the clinic where patients are treated or the court that ordered the initial detention, as shown by Querengässer et al. (23), the reliance on hierarchical linear regression analyses to evaluate the predictive value of criminal history on cognitive performance may not capture the full complexity of these relationships. Alternative statistical models that account for non-linear interactions, latent variables, and individual differences in treatment response could provide a more nuanced understanding of the data. Given the small sample size of this study, future research should aim to include a larger cohort to enhance the generalizability of findings and to allow for more definitive conclusions about the relationships between neuropsychological impairment, substance use, and criminal behavior.

For the future, it is imperative that research expands upon these foundations through longitudinal studies that track changes over time with treatment interventions. Moreover, incorporating a wider array of neuropsychological assessments and considering the effects of treatment modalities on cognitive outcomes will be crucial in developing more effective rehabilitation programs tailored to the specific needs of this complex population. Such studies could enhance our understanding of the specific cognitive deficits that are most salient for criminal behavior and recidivism.

## 5 Conclusion

In conclusion, this study advances our understanding of the cognitive dimensions of impulsivity and criminality in substance-using forensic populations. The investigation itself reveals a complex and somewhat equivocal set of findings. While higher levels of impulsivity were confirmed compared to the general population, consistent with prior research, the hypothesized correlations between broader neuropsychological functions and criminal histories were not robustly supported across a wide range of cognitive tasks. Targeting this relationship, only tasks related to planning and sustained attention, showing clearer associations. This inconsistency suggests that while substance use disorders are associated with increased impulsivity, their impact on other cognitive functions might be more complex and less uniform than traditionally assumed. This study highlights the need for a multidimensional assessment strategy that integrates impulsivity metrics with a broader range of neuropsychological tests to enhance predictive accuracy and treatment personalization. Targeted cognitive assessments could be pivotal in refining therapeutic and rehabilitation strategies, tailoring them more closely to individual cognitive deficits and criminogenic needs. More nuanced research approaches, an extensive participant base and varied neuropsychological assessments will be essential to disentangle the cognitive and behavioral profiles characteristic of this group, thereby improving the clinical and forensic outcomes.

## Data Availability

The original contributions presented in the study are included in the article/supplementary material. Further inquiries can be directed to the corresponding author.
